# Diabetes Mellitus and Associated Factors in Slovakia: Results from the European Health Interview Survey 2009, 2014, and 2019

**DOI:** 10.3390/nu13072156

**Published:** 2021-06-23

**Authors:** Nour Mahrouseh, Carlos Alexandre Soares Andrade, Nóra Kovács, Diana Wangeshi Njuguna, Orsolya Varga

**Affiliations:** 1 Department of Public Health and Epidemiology, Faculty of Medicine, University of Debrecen, 4032 Debrecen, Hungary; nour.mahrouseh@med.unideb.hu (N.M.); soares.andrade@med.unideb.hu (C.A.S.A.); kovacs.nora@med.unideb.hu (N.K.); diana.njuguna@med.unideb.hu (D.W.N.); 2 Office for Supported Research Groups, Eötvös Loránd Research Network, 1052 Budapest, Hungary

**Keywords:** EHIS, diabetes, disease burden, European Union, policies

## Abstract

Diabetes mellitus (DM) is a high-risk non-communicable disease with an emerging burden for the European Union (EU) member states in the past decades. The unfavorable trend of the burden is striking compared to the declining disease burden due to cardiovascular diseases or stagnation of neoplasms. The goal of this study is to describe the temporal changes of diabetes in the adult population of Slovakia through the three European Health Interview Survey (EHIS) waves and to assess the association between DM and socioeconomic and/or lifestyle characteristics. These cross-sectional studies were carried out using microdata derived from Slovakia’s EHISs conducted in the years 2009 (*n* = 4972), 2014 (*n* = 5490), and 2019 (*n* = 5527). The DM variable was compared to the independent variables such as sociodemographic and lifestyle characteristics including dietary patterns and physical activity. DM prevalence for the EHIS in 2009, 2014, and 2019 were 6.1%, 8.2%, and 9.8%, respectively. In bivariate analysis, the relationship between DM and age, education level, job status, BMI, walking for at least 10 min, and physical activity was significant in the three EHISs. In 2014 and 2019, there was an inverse association between the risk of DM and walking regularly. There was no association between the frequency of eating fruits or vegetables and DM, with the exception of 2009, where a negative association between eating vegetables one to six times a week and DM was observed. Present health policies and activities in Slovakia were unable to reverse the increasing DM burden, indicating that a more systematic approach is needed. Complex policy strategies and legislative measures must be developed and implemented at both the national and EU levels.

## 1. Introduction

Diabetes mellitus (DM) and its complications rank high in the global list of diseases with high burden values [1]. DM is one of the four most common chronic non-communicable diseases (NCDs), which occurs due to inadequate insulin production in the pancreas, or when the body does not use its insulin production properly, which impact blood sugar regulation. Uncontrolled DM can lead to several systemic complications in the body. According to the World Health Organization (WHO), the three major types of DM are type 1 diabetes mellitus, type 2 diabetes mellitus (T2DM), and gestational diabetes. Among these, the T2DM is by far the most common, accounting for 90% of total DM cases. With regard to its frequency and preventability, policies and strategies for the prevention of DM are mainly tackling diabetes T2DM risk factors [2,3]. The European Union (EU) member states are especially facing an increasing burden of DM; projections for 2030 and 2045 show that the prevalence of DM among adults will increase to 50.48% and 50.51%, respectively [4]. Such a trend is very unfavorable compared to the two other major NCDs—cardiovascular diseases and neoplasms. According to the Global Burden of Disease (GBD) database, the trend of DALYs (age-standardized rate per 100,000) due to cardiovascular diseases showed a considerable decline from 8477.91 in 1990 to 5779.75 in 2019. Neoplasms had a plateau in trend with modest change on DALYs from 5752.73 in 1990 to 6022.41 in 2019. In the same time period, DM was reported to show a significant increase of burden, from 741.63 to 1098.57 [1].

In European countries, efforts have been made to tackle DM since the establishment of the St. Vincent Declaration in 1989. Although some political initiatives by the European Parliament were taken [5], in the last 20 years, DM has been mostly addressed as one of the NCDs—for example, the Action Plan for the Prevention and Control of Non-communicable Diseases in the WHO European Region [6].

However, according to Article 168 of the Treaty on the Functioning of the European Union, the member states have the primary role of organizing the healthcare services; the EU provides support and funds for prevention and research to reduce the burden due to DM in many ways [7]—for instance, by facilitating the production of comparable datasets for the identification and adoption of effective health policy measures. The European Health Interview Survey (EHIS) is the most significant health data collection instrument. It provides information on the health status, health determinants and healthcare services facilitating data comparability between the member states [8]. These comparable data allow stakeholders to develop and select tailored responses targeting the causal factors of NCDs.

According to the WHO, the onset of type 2 diabetes can be delayed by maintaining a healthy body weight, being physically active, eating a healthy diet and avoiding tobacco use [9]. Poor diet is one of the major issues implicated in the incidence of DM. High intake of foods with a high quantity of sugar, such sweetened beverages, can lead to worsened DALYs in countries of the EU. Moreover, the suboptimal consumption of whole grains, nuts, fruit, fish and legumes have a negative impact on the burden of DM [10]. Increased physical activity, exercise or training, and reduced sedentary lifestyle are also behaviors needed to avoid the occurrence of DM [11].

In addition to lifestyle related factors, socioeconomic factors also influence the development of DM. Population-based studies have reported an association with older age, lower socioeconomic status, and a lower level of education [12]. The burden due to DM varies significantly among member states, and the diversity is present at a subnational and regional level [13,14]. Thus, individual-based strategies addressing socioeconomic disadvantages also seem necessary for DM prevention [15].

This study aims to describe the temporal changes of DM in the adult population of Slovakia through the three EHIS waves, and to assess the association between DM and socioeconomic and/or lifestyle characteristics. This study shows important results from an ongoing project analyzing data from the three EHIS waves with a focus on DM in the EU.

## 2. Methods

Cross-sectional studies were carried out using microdata derived from Slovakia’s EHISs conducted in 2009 (*n* = 4972), 2014 (*n* = 5490), and 2019 (*n* = 5527). The microdata was obtained from Eurostat for European Health Interview survey 2009 and 2014, and The Statistical Office of the Slovak Republic for European Health Interview Survey 2019. These samples are representative of the Slovakian adult population (aged 15 years or over) residing in private households. The three surveys included different participants. The EHIS data collection method differs between countries, which may include face-to-face interviews, telephone interviews, postal, web interviews or a combination of these methods. Self-administered questionnaires were also applied for some questions; through papers, the Internet, or both. According to the quality report of wave 2 in Slovakia, face-to-face interviews and self-administered paper-based questionnaires were used. The sampling frame varied by countries as some used population registers, dwelling registers, population censuses, and others. Slovakia’s sampling frame was drawn from the dwelling register. Some countries used proxy interviews; in Slovakia, proxy interviews were not applied. Details of the methodology and sampling are reported by the European Commission [8]. Our study variables were based on questions consequently asked in the 2009, 2014, and 2019 EHISs. Respondents who answered “yes” to the question: “During the past 12 months, have you had diabetes?” were considered in the group with DM, including any types of diabetes mellitus. Self-reporting sociodemographic and lifestyle characteristics were analyzed as independent variables; a definition of each variable as derived from the survey is available in Supplementary.

Demographic and socioeconomic characteristics included sex, age (15 to 44, 45 to 64, and 65 and above), regions based on level 2 of nomenclature of territorial units for statistics (NUTS2) of Slovakia (Bratislavský kraj, Západné Slovensko, Stredné Slovensko, and Východné Slovensko), degree of urbanization (cities, towns and suburbs and rural areas), education level (less than primary/primary education, secondary education and higher education), labor status (employed, unemployed and others).

The included lifestyle variables were body mass index (BMI, kg/m^2^) (<18.5, 18.5–24.9, 25–29.9 and ≥30), frequency of walking for transportation purposes (to get to and from places) at least 10 min per day (everyday, one to six days, and never), physical activity per week (two days and more, one day per week and never), the frequency of eating fruits and frequency of eating vegetables per week (one or more per day, one to six times a week, and less than once a week and never).

The distribution of the variables was described and compared within surveys and for diabetic and nondiabetic respondents. Proportions were used as descriptive statistics. The estimated prevalence of DM for each year was based on the study sample. To perform bivariate comparisons, Pearson chi-square test was used to analyze the association between the study variables and DM. A multivariable unconditional logistic regression model was conducted, including variables which were statistically significant in the bivariate analysis, and variables that were not statistically significant, but were of interest from an epidemiological perspective. The regression results are shown as odds ratios (ORs) with 95% confidence interval (CIs). We used the three datasets separately for analysis. Sampling weights were available in the database; svy function in Stata was used to preserve the EHIS survey weighting only in the multivariable analysis. Statistical analyses were performed by using STATA IC version 13.0 software.

## 3. Results

DM prevalence for the 2009 EHIS was 6.1%, 8.2% for 2014, and 9.8% for 2019. The distribution by numbers of the study population by DM occurrence according to demographic, socioeconomic variables, and lifestyle are shown in Table 1. The distribution by numbers and relative frequencies of the study population divided by the presence of DM according to demographic, socioeconomic variables, and lifestyle are shown in Table S1 of the Supplementary File.

The results of the bivariate analysis showed that the percentage of individuals who had DM differed by gender (*p*-value < 0.05) in 2009; female respondents had higher percentages in the diabetes group than males. Frequencies were significantly different by age groups in 2009, 2014, and 2019: diabetic respondents belonged to older age categories of 65 and older—3.59%, 4.81%, and 6.06%, respectively. It is observed that respondents with secondary education were more affected by DM than any other education levels and the relation between DM and education level is significant. The degree of urbanization presented a significant relationship with DM only in 2009 and 2014; the respondents who had DM and lived in the rural areas were higher in 2009. In 2014, the most individuals with DM lived in towns and suburbs. The occurrence of DM differed by labor status; respondents who were diabetic were higher in other groups (e.g., students, pensioners) of labor status. As seen by the frequencies in the cross-tabulated Table 1, there is a significant relationship between the presence of DM and BMI in 2009, 2014, and 2019. The majority of diabetic individuals belonged to the overweight or obesity group. Individuals with DM were higher in the BMI group between 25 to 29.9 in 2009 and 2014, but in 2019, the frequency of diabetes was higher in the BMI group ≥30. The frequency of having DM differed by the number of days walking for at least 10 min per week. Of the total number of respondents, most of the diabetic individuals in 2009 and 2014 walked to get to and from places one to six times per week for 10 min. Walking every day for transportation purposes was higher and less diabetic individuals never walked in 2009 and 2014. In 2019, respondents with DM had a higher frequency for walking for transportation for 10 min every day than walking one to six times per week for 10 min or never groups. A significant relationship was shown between the presence of DM and physical activity categories in 2009, 2014, and 2019. Higher frequencies of diabetic individuals were found in the group of individuals who never performed any kind of physical activity per week in 2009, 2014, and 2019. The relationship between the consumption of fruits and vegetables and the presence of DM was significant only in 2009. Diabetic respondents had the highest frequency of eating one or more fruits and vegetables per day in 2009 than eating one to six times a week or less than once a week or never.

The results of the multivariable model are shown in Table 2. The results indicated that females in 2019 were 27% less likely to have DM compared to males (OR 0.77; 95% CI 0.62 to 0.95). Age groups of 15 to 44 and 45 to 64 had a negative association to DM compared to the reference age group (65 and above) in all three surveys. The degree of urbanization was not associated with the presence of DM. Primary or less than primary education was positively associated with having DM in 2019 (OR 3.25; 95% CI 1.12 to 9.46) compared to respondents who were in the higher education category. Employment as a labor status had significant lower likelihood of DM, compared to the reference group of other labor status (OR 0.40; 95% CI 0.27–0.60) (OR 0.35; 95% CI 0.25–0.49) (OR 0.36; 95% CI 0.25 to 0.50) in the years of 2009, 2014 and 2019. Unemployment (OR 0.56; 95% CI 0.33 to 0.95) presented lower probability of having DM compared to reference group of other labor status, only in 2014.

People with BMI of 30 or higher had a greater probability of developing DM in 2009 and 2019 as compared to the overweight group (BMI 25 to 29.9), and accordingly, lower BMI (<18.5 to 24.9) was associated with a low probability of having DM in 2009 (OR 0.59; 95% CI 0.41 to 0.84), 2014 (OR 0.57; 95% CI 0.43 to 0.76), 2019 (OR 0.65; 95% CI 0.49 to 0.88), respectively. Regarding physical activity and movement, a negative association between the risk of DM and walking to get to and from places for at least 10 min every day (OR 0.67;95% CI 0.49 to 0.92) (OR 0.57; 95% CI 0.42 to 0.77) or at least one to six times per week (OR 0.70; 95% CI 0.52 to 0.96) (OR 0.69; 95% CI 0.50 to 0.94) compared to our reference category of “never” was found in the years of 2014 and 2019, respectively. In 2014, a lack of physical activity, “never”, increased the probability of DM (OR 2.55; 95% 1.02 to 6.37). There was no association between the frequency of eating fruits and the presence of DM. The frequency of eating vegetables one to six times per week compared to our reference category of one and more a day decreased the risk of DM presence in the year 2009 (OR 0.66; 95% CI 0.48 to 0.92). There was no significant association regarding regions of Slovakia (data from 2019, exclusively).

## 4. Discussion

To the best of our knowledge, this is the first study analyzing DM burden throughout the three waves of EHIS. Our study presents nationwide, representative data on an adult population covering basic health monitoring indicators, establishing the required baseline data for future evaluation. According to our analysis, the prevalence of DM patients has shifted upwards from 2009 to 2019. This increase is similar to the increase of prevalence in most of the EU member states and the EU average [16].

The results showed that the degree of urbanization was not associated with the risk of DM, contrary to results from a meta-analysis, which considered living in cities would elevate the risk of DM [17]. Socioeconomic factors such as lower education attainment level and labor status may function as factors due to various disparities in the population [18,19]. Results from Denmark and other European countries demonstrate that individuals who are less educated are prone to develop DM than those without. Employment is inversely linked to DM in the three waves; studies have found that individuals with DM exit labor earlier, in addition to reducing their quality of work [20]. Studies suggest that the work environment, type of work and working hours increase the risk of DM [21,22,23].

Dietary habits have shifted in all EU countries to a “Westernized” diet based on the globalization of food production and distribution, which is most striking in the Mediterranean countries [24]. The vegetable intake in Europe increased by approximately 20% from the middle of the last century until 2006. Historically, countries of southern Europe reported the highest vegetable intake (double other European regions), but in the beginning of this century, it has started to decrease. Fruit consumption has increased across Europe over the past 60 years, in line with vegetable consumption. Market sales of fruit increased until the beginning of the 21st century, followed by a slow decline. At a regional level, the most significant growth was observed in Northern European countries, where it has been slowly declining in recent years [25].

Our study has supported the already established evidence of the association between obesity and overweight with higher DM risk, which was revealed significantly through the three waves. Fruit and vegetable intake is often at the center of health policies tackling DM. In some prospective studies, fruit and vegetable consumption was found to reduce the risk of DM [26,27]. However, in our cross-sectional analyses, their consumption did not show a consistent association with DM occurrence. People with DM may consume similar amounts of fruits and vegetables as individuals without DM following healthcare recommendations [6,7]. This contradiction is not surprising; the lack of a clear link between total vegetable and fruit consumption and the incidence of T2DM was already reported by a meta-analysis [28].

In high-income countries such as Slovakia, DM occurrence may be more associated with obesity and physical inactivity than other socioeconomic factors and urbanization. This might be caused by diet transitioning towards Westernized diet in recent years. The dietary quality of a country may depend on interrelated factors including traditional food patterns, local food availability, the food supply chain, and food policies.

Sedentary lifestyle, decreased physical activity, is more so than an unhealthy diet considered to be associated directly or indirectly with DM. A study using data from Sport and Physical Activity EU Special Eurobarometers reported that sedentary behavior became more prevalent from 2005 to 2017 in the EU member states (except for Finland) and this increasing prevalence occurred in the total population, and men and women separately. The higher prevalence of sedentary lifestyle was observed among men than women, except for Bulgaria, Estonia, Hungary, Latvia and Lithuania [29].

Based on our results, walking as part of active transportation was a protective factor for DM, which aligns with results from a dose–response meta-analysis; walking up to two to three hours per week reduces the risk of DM, but above these levels, there is no reduction in the risk [30]. Individuals who had a sedentary lifestyle were associated with DM. Clinical trials and cohorts found that both aerobic and resistance exercise have an inverse association with DM risk [31]. Meta-analysis suggests a greater risk of DM associated with a large duration of sedentary behavior [32].

Human studies have supported that DM can be delayed or managed integrating a multi-component approach including the main affecting socioeconomic and lifestyle factors through regulating food intake, behavioral changes, and physical activity. A recent randomized controlled trial found that intensive lifestyle interventions resulted in significant weight loss over 12 months, and that more than 60% of participants experienced the remission of diabetes and 30% achieved normoglycemia [33].

Although Slovakia has already established legislative efforts in these domains [34,35]—which include a policy on the organization of sport in educational settings and promoting sport in younger ages, as well as a national action plan to promote sport and physical activity, several nutritional and labeling policies targeting obesity and improving health, and a national DM plan—the burden of DM is still high [36]. However, the experience with policy interventions at the population level is controversial. While population-based interventions are often followed by some successes, these do not necessarily translate into long-term reductions in disease burden. A systematic review, for example, about regulatory interventions targeting population nutrition found that some “isolated regulatory interventions” may have a positive impact on intermediate outcomes, but this change has not reached clinically significant levels—e.g., having such an impact on food intake that can result in reduced incidence of obesity or NCDs [37]. Similarly, another systematic review has not found evidence of the impact of any of the studied interventions on the prevalence of overweight, obesity, or T2DM [38]. Simulation studies project that a network of interventions is needed to achieve the targets in disease burden reduction. In a model with all potential interventions incorporated, the population risk ratios could be reduced both for obesity and T2DM [39].

The major limitation of this study is that, due to the cross-sectional design, causal relationship between DM and risk factors cannot be established. In our analysis, the employment position was not considered. Regions of level 2 of nomenclature of territorial units for statistics (NUTS2) were only available in 2019; thus, the association between DM and living in different regions of Slovkia was studied in 2019 exclusively. EHIS is a self-reported survey; all answers were subjective, which may affect the accuracy and reliability of the reported data and estimated associations. Additionally, due to the homogenous category of DM that was used in the EHIS waves, the different types of DM were not distinguished in our analysis, although the background pathologies of each type are different.

## 5. Conclusions

Lifestyle characteristics such as dietary habits of eating fruit and vegetables were not associated with DM, but results from 2009 demonstrated that eating vegetables several times a week may reduce the risk of DM and movement for at least 10 min, as walking may prevent DM. However, more lifestyle characteristics and socioeconomic conditions should be studied to evaluate their role in the increased prevalence in Slovakia and in comparison to other EU countries. In the EU, the member states have a leading role in combating DM and its risk factors, to make legislation and provide healthcare services. In Slovakia, existing health policies and actions could not reverse the gradually growing DM burden, indicating that a more systematic approach should be adopted. In conclusion, to achieve improvement, creating and implementing complex policy initiatives and legislative measures seem unavoidable, both at national and EU levels.

## Figures and Tables

**Table 1:** Distribution of the study population.

Variable	Category	EHIS 2009			EHIS 2014			EHIS 2019		
		With Diabetes *n*	Without Diabetes *n*	*p*-Value	With Diabetes *n*	Without Diabetes *n*	*p*-Value	With Diabetes *n*	Without Diabetes *n*	*p*-Value
Sex	Male	132	2257	0.022	184	2270	0.098	233	2087	0.799
	Female	183	2392		265	2771		317	2898	
Age	15 to 44	Below 20	2689	<0.001	Between 20 and 49	2467	<0.001	Between 20 and 49	1933	<0.001
	45 to 64	118	1427		149	1673		180	1843	
	65 and older	178	533		264	901		335	1209	
Region *	Bratislavský kraj							Between 20 and 49	621	0.104
	Západné Slovensko							194	1674	
	Stredné Slovensko							138	1220	
	Východné Slovensko							160	1470	
Degree of urbanization	Cities	54	1109	0.006	92	1415	<0.001	113	1127	0.342
	Towns and suburbs	120	1445		196	2045		178	1721	
	Rural areas	141	2095		161	1581		249	2137	
Education level	Primary/less than primary education	Below 20	68	0.049	Below 20	Between 20 and 49	<0.001	Below 20	Below 20	<0.001
	Secondary education	270	3724		405	4009		482	3930	
	Higher education	Between 20 and 49	857		Between 20 and 49	991		51	1027	
Labor activity status	Employed	66	2730	<0.001	59	2393	<0.001	86	2503	<0.001
	Unemployed	Below 20	299		Below 20	476		Below 20	263	
	Others	241	1620		372	2172		437	2219	
	<18.5	Below 20	154	<0.001	Below 20	131	<0.001	Below 20	102	<0.001
BMI (kg/m^2^)	18.5 to 24.9	60	2212		88	2206		81	1883	
	25 to 29.9	130	1529		222	1888		205	1949	
	≥30	109	598		138	816		243	971	
Frequency of walking for transportation purposes for at least 10 min continuously per week	Everyday	113	2049	<0.001	176	2381	<0.001	245	2864	<0.001
	One to six days	116	1989		188	2114		195	1690	
	Never	65	429		85	546		99	422	
Physical activity	2 Days and more	138	2996	<0.001	Between 20 and 29	1503	<0.001	53	1406	<0.001
	One day per week	Between 20 and 49	259		below 20	230		Below 20	200	
	Never	143	1132		399	3308		476	3378	
Frequency of eating fruits	One or more per day	230	2953	0.001	210	2402	0.185	281	2682	0.701
	One to six times a week	69	1492		204	2353		226	1993	
	Less than once a week and never	Below 20	193		Between 20 and 49	286		Between 20 and 49	308	
Frequency of eating vegetables or salad	Once and more a day	191	2366	0.001	192	2220	0.843	248	2363	0.693
	One to six times a week	104	2023		230	2539		255	2333	
	Less than a week and never	Below 20	247		Between 20 and 49	282		Between 20 and 49	287	

Legend: “below 20” represents below 20 observations in the cell, “between 20 and 45” represents observations between 20 and 45 in the cell. “Below 20”, “between 20 and 45” are used according to the database guideline of statistical disclosure control. * Sorted by gross domestic product (GDP) at current market prices by NUTS 2 regions of Slovakia in Euros (€) per inhabitant, Bratislavský kraj 39,700 € per inhabitant, Západné Slovensko 15,800 € per inhabitant, Stredné Slovensko 14,100 € per inhabitant, Východné Slovensko 12,200 € per inhabitant. BMI body mass index (kg/m^2^).

**Table 2:** Factors associated with diabetes Variable.

Variable	Category	EHIS 2009	EHIS 2014	EHIS 2019
		OR (95% CI)	OR (95% CI)	OR (95% CI)
Sex (ref: males)	Female	0.85 (0.64–1.14)	0.91 (0.73–1.13)	0.77 (0.62–0.95) *
Age (ref: 65 and older)	15 to 44	0.06 (0.04–0.10) *	0.14 (0.09–0.21) *	0.12 (0.07–0.19) *
	45 to 64	0.46 (0.32–0.65) *	0.54 (0.41–0.71) *	0.71 (0.54–0.95) *
Region (ref: Bratislavský kraj)	Západné Slovensko			0.83 (0.52–1.31)
	Stredné Slovensko			0.90 (0.57–1.44)
	Východné Slovensko			0.92 (0.58–1.46)
Degree of urbanization (ref: rural areas)	Cities	0.82 (0.55–1.22)	0.82 (0.60–1.11)	1.19 (0.87–1.63)
	Towns and suburbs	1.19 (0.87–1.63)	1.14 (0.89–1.45)	1.03 (0.82–1.30)
Education level (ref: higher education)	Primary/less than primary education	0.38 (0.10–1.43)	1.23 (0.47–3.22)	3.25 (1.12–9.46) *
	Secondary education	0.71 (0.48–1.06)	1.29 (0.89–1.88)	1.36 (0.97–1.92)
Labor status (ref: others)	Employed	0.40 (0.27–0.60) *	0.35 (0.25–0.49) *	0.36 (0.25–0.50) *
	Unemployed	0.72 (0.33–1.56)	0.56 (0.33–0.95) *	0.65 (0.36–1.18)
BMI (kg/m^2^) (ref: 25 to 29.9)	<18.5	0.64 (0.15–2.69)	0.08 (0.01–0.56)	1.04 (0.34–3.13)
	18.5 to 24.9	0.59 (0.41–0.84) *	0.57 (0.43–0.76) *	0.65 (0.49–0.88) *
	≥30	1.81 (1.30–2.52) *	1.11 (0.86–1.44)	2.04 (1.62–2.57) *
Frequency of walking for transportation purposes for at least 10 min continuously per week (ref: never)	Everyday	0.76 (0.51–1.14)	0.67 (0.49–0.92) *	0.57 (0.42–0.77) *
	One to six days	0.76 (0.51–1.13)	0.70 (0.52–0.96) *	0.69 (0.50–0.94) *
Physical activity (ref: one day per week)	Two days and more	0.65 (0.38–1.11)	1.53 (0.59–3.96)	0.63 (0.31–1.31)
	Neve	0.95 (0.55–1.64)	2.55 (1.02–6.37) *	1.43 (0.73–2.81)
Frequency of eating fruits (ref: one or more per day)	One to six times a week	0.96 (0.67–1.39)	0.98 (0.73–1.32)	1.10 (0.81–1.51)
	Less than once a week and never	1.18 (0.55–2.51)	1.54 (0.87–2.71)	1.07 (0.59–1.92)
Frequency of eating vegetables or salad (ref: one or more per day)	One to six times a week	0.66 (0.48–0.92) *	1.07 (0.80–1.44)	0.82 (0.60–1.11)
	Less than a week and never	0.67 (0.34–1.30)	0.79 (0.41–1.52)	0.80(0.46–1.39)

Legend: * significant association (*p* < 0.05) between with diabetes and without diabetes in regression model. BMI body mass index (kg/m^2^).

## Data Availability

Microdata of EHIS 2009 and 2014 are available via Eurostat upon request. Microdata of EHIS 2019 is available via Statistical Office of the Slovak Republic upon request.

## Supplementary Materials

The following are available online at https://www.mdpi.com/article/10.3390/nu13072156/s1. Table S1: Distribution of the study population. Definitions of the variables that were used in the study.

## Source of the Data

This study is based on data from Eurostat, European Health Interview survey 2009, 2014, and Statistical Office of the Slovak Republic, European Health Interview Survey, 2019.
