# Supplementary material for: Diabetes Mellitus and Associated Factors in Slovakia: Results from the European Health Interview Survey 2009, 2014, and 2019
Source: Nutrients. 2021 Jun 23;13(7):2156. doi: 10.3390/nu13072156 (PMC8308286; doi:10.3390/nu13072156)
Supplement: Supplementary file 1 [file nutrients-13-02156-s001.zip › nutrients-1242493-supplementary.pdf]

Title: Diabetes mellitus and associated factors in Slovakia: Results from the European Health Interview Survey 2009, 2014 and 2019

Authors: Nour Mahrouseh, Carlos Alexandre Soares Andrade, Nóra Kovács, Diana Wangeshi Njuguna and Orsolya Varga

Supplementary file 1:

Definitions of the variables that were used in our study

- Sex was based on the question "Sex". The self-reported answers were divided into two categories, male and female.
- Age was based on the question "Age of the person interviewed at the moment of interview" in 2009, and the question "Age of respondent in completed years at the time of the interview" in 2014, and "Year of birth" in 2019. The self-reported answers were divided into three categories (15-44, 45-64, 65 and older).
- Region was based on the question "Region of residence". The self-reported answers were according to NUTS at 2-digit level.
- Degree of urbanization was based on the question "Degree of urbanisation". The self-reported answers were divided into three categories. Cities: based on self-reported answers (densely-populated area) in 2009 and 2014, and self-reported answers (cities) in 2019. Towns and suburbs: based on self-reported answers (intermediate-populated area) in 2009 and 2014, and self-reported answers (Towns and suburbs) in 2019. Rural areas: based on self-reported answers (thinly-populated area) in 2009 and 2014, and self-reported answers (rural areas) in 2019.
- Educational level was based on the question "What is the highest education leaving certificate, diploma or education degree you have obtained?". The self-reported answers were divided into three categories. Primary or less than primary education: based on self-reported answers (no formal education or below, primary education, lower secondary education) in 2009, 2014 and 2019. Secondary education: based on self-reported answers (upper secondary education, post-secondary but non-tertiary education tertiary education; short-cycle) in 2009, 2014 and 2019. Higher education: based on self-reported answers (first stage of tertiary education, second stage of tertiary education) in 2009, and self-reported answers (bachelor level or equivalent, master level or equivalent, doctoral level or equivalent) in 2014 and 2019.
- Labour status was based on the question "How would you define your current labour status?". The self-reported answers were divided into three categories. Employed: based on the self-reported answer (working for pay or profit (including unpaid work for a family business or holding, including an apprenticeship or paid traineeship, including currently not at work due to maternity, parental, sick leave or holidays)) in 2009, self-reported answer (carries out a job or profession, including unpaid work for a family business or holding, an apprenticeship or paid traineeship, etc.) in 2014 and self-reported answer (employed) in 2019. Unemployed: based on the self-reported answer (unemployed) in 2009, 2014 and 2019. Others: based on the self-reported answers (pupil, student, further training, unpaid work experience, in retirement or early retirement or has given up business, permanently disabled, in compulsory military or community service, fulfilling domestic tasks, other) in 2009, self-reported answers (pupil, student, further training, unpaid work experience, In retirement or early retirement or has given up business, permanently disabled, in compulsory military or community service, fulfilling domestic tasks and other inactive person) in 2014, and self-

reported answers (retired, Unable to work due to longstanding health problems, student, pupil, fulfilling domestic tasks, compulsory military or civilian service, and other) in 2019.

- BMI was calculated based on two questions (How tall are you without shoes?) and (How much do you weigh without clothes and shoes?). The self-reported answers were available in centimeters (cm) and kilograms(kg), respectively, calculated based on the formula “kg/m^2” and divided into three categories (<25 , 25-29.9 and ≥30).
- Frequency of walking for at least 10 minutes continuously for transportation purposes (to get to and from places) per week was based on the question “In a typical week, on how many days do you walk for at least 10 minutes continuously in order to get to and from places?” answers of self-reported number of days were divided into three categories. Everyday: self-reported number of days equals to or larger than 7 days per week. 1 to 6 times a week: self-reported number of days between 1 to 6 days per week. Less than once a week and never: self-reported number of days equals to 0 days per week.
- Physical activity was based on merging two questions “During the past 7 days, on how many days did you do vigorous physical activities?” and “During the past 7 days, on how many days did you do moderate physical activities?”. The self-reported answers were divided into three categories. 2 days and more: self-reported number of days equals to or larger than 2 days per week. Never: self-reported number of days equals 0 days per week, one day per week: self-reported number of days equals to 1 day per week.
- Frequency of eating fruits was based the question “How often do you eat fruits (excluding juice)?”. The self-reported answers were divided into three categories. One and more per day based on self-reported answers (twice or more a day and once a day) in 2009 and self-reported answers (once or more a day) in 2014 and 2019. 1 to 6 times a week based on self-reported answers (less than once a day but at least 4 times a week and less than 4 times a week and but at least once a week). Less than once a week and never: based on the self-reported answers (less than once a week and never).
- Frequency of eating vegetables or salad was based the question “How often do you eat vegetables or salad (excluding juice and potatoes)?”. The self-reported answers were divided into three categories. One and more per day based on self-reported answers (twice or more a day and once a day) in 2009 and self-reported answers (once or more a day) in 2014 and 2019. 1 to 6 times a week based on self-reported answers (less than once a day but at least 4 times a week, less than 4 times a week and but at least once a week). Less than once a week and never: based on the self-reported answers (less than once a week and Never).

Sources:

European Health Interview Survey (EHIS wave 1) guidelines 2010

European Health Interview Survey (EHIS wave 2) Methodological manual : 2013 edition

European Health Interview Survey (EHIS wave 3) Methodological manual : 2018 edition

Table S1 of the supplementary file. Distribution of the study population

Variable	Category	EHIS 2009		EHIS 2014		EHIS 2019	
		With diabetes N(%)	Without diabetes N(%)	With diabetes N(%)	Without diabetes N(%)	With diabetes N(%)	Without diabetes N(%)
sex[a]	Male	132(2.66%)	2,257(45.47%)	184(3.35%)	2,270(41.35%)	233(4.04%)	2,087(37.77%)

	Female	183(3.69%)	2,392(48.19%)	265(4.83%)	2,771(50.47%)	317(5.74%)	2,898(52.45%)
Age[a,b,c]	15-44	Below 20	2,689	Between 20-49	2,467	Between 20-49	1,933
	45-64	118(2.38%)	1,427(28.75%)	149(2.71%)	1,673(30.47%)	180(3.26%)	1,843(33.36%)
	65 and older	178(3.59%)	533(10.74%)	264(4.81%)	901(16.41%)	335(6.06%)	1,209(21.88%)
Region*	Bratislavský kraj					Between 20-49	621
	Západné Slovensko					194(3.51%)	1,674(30.30%)
	Stredné Slovensko					138(2.50%)	1,220(22.08%)
	Východné Slovensko					160(2.90%)	1,470(26.61%)
Degree of urbanization[a,b]	Cities	54(1.09%)	1,109(22.34%)	92(1.68%)	1,415(25.77%)	113(2.05%)	1,127(20.40%)
	Towns and suburbs	120(2.42%)	1,445(29.11%)	196(3.57%)	2,045(37.25%)	178(3.22%)	1,721(31.15%)
	Rural areas	141(2.84%)	2,095(42.20%)	161(2.93%)	1,581(28.80%)	249(4.51%)	2,137(38.68%)
Educational level[a,b,c]	Primary Lless than primary education	Below 20	68	Below 20	Between 20-49	Below 20	Below 20
	Secondary e ducation	270(5.44%)	3,724(75.02%)	405(7.38%)	4,009(73.02%)	482(8.72%)	3,930(71.13%)
	Higher education	Between 20-49	857(17.26%)	Between 20-49	991	51(0.92%)	1,027(18.59%)
Labour activity status[a,b,c]	Employed	66(1.33%)	2,730(55%)	59(1.07%)	2,393(43.59%)	86(1.56%)	2,503(45.30%)
	Unemployed	Below 20	299	Below 20	476	Below 20	263
	Others	241(4.85%)	1,620(32.63%)	372(6.78%)	2,172(39.56%)	437(7.91%)	2,219(40.16%)
BMI (kg/m^2)[a,b,c]	<18.5	Below 20	154	Below 20	131	Below 20	102
	18.5-24.9	60(1.25%)	2,212(46.14%)	88(1.60%)	2,206(40.18%)	81(1.49%)	1,883(34.62%)
	25-29.9	130(2.71%)	1,529(31.89%)	222(4.04%)	1,888(34.39%)	205(3.77%)	1,949(35.83%)
	≥30	109(2.27%)	598(12.47%)	138(2.51%)	816(14.86%)	243(4.47%)	971(17.85%)
Frequency of walking for transportation purposes for at least 10 minutes	Everyday	113(2.37%)	2,049(43.04%)	176(3.21%)	2,381(43.37%)	245(4.44%)	2,864(51.93%)
	1 to 6 days	116(2.44%)	1,989(41.78%)	188(3.42%)	2,114(38.51%)	195(3.54%)	1,690(30.64%)

continuously per week[a,b,c]	Never	65(1.37%)	429(9.01%)	85(1.55%)	546(9.95%)	99(1.80%)	422(7.65%)
Physical activity[a,b,c]	2 Days and more	138(2.94%)	2,996(63.89%)	Between 20-29	1,503	53(0.96%)	1,406(25.45%)
	One day per week	Between 20-49	259	below 20	230	Below 20	200
	Never	143(3.05%)	1,132(24.14%)	399(7.27%)	3,308(60.26%)	476(8.62%)	3,378(61.15%)
Frequency of eating fruits[a]	One and more per day	230(4.64%)	2,953(59.63%)	210(3.83%)	2,402(43.75%)	281(5.09%)	2,682(48.56%)
	1 to 6 times a week	69(1.39%)	1,492(30.13%)	204(3.72%)	2,353(42.86%)	226(4.09%)	1,993(36.09%)
	Less than once a week and never	Below 20	193	Below 20-49	286	Between 20-49	308
Frequency of eating vegetables or salad[a]	Once and more a day	191(3.86%)	2,366(47.80%)	192(3.50%)	2,220(40.44%)	248 (4.49%)	2,363(42.78%)
	1 to 6 time a week	104(2.10%)	2,023(40.87%)	230(4.19%)	2,539(46.25%)	255 (4.62%)	2,333(42.24%)
	Less than a week and never	Below 20	247	Between 20-49	282	Between 20-49	287

Legend : a, Significant association ($p<0.05$) between with diabetes and without diabetes in 2009. b, Significant association ($p<0.05$) between with diabetes and without diabetes in 2014. c, Significant association ($p<0.05$) between with diabetes and without diabetes in 2019, "Below 20 "represents below 20 observations in the cell, "Between 20-45" represents observations between 20-45 in the cell. "Below 20", "Between 20-45"and missing values are used in according to the database guidelines in terms of statistical disclosure control. * Sorted by gross domestic product (GDP) at current market prices by NUTS 2 regions of Slovakia in Euros (€) per inhabitant, Bratislavský kraj 39,700 € per inhabitant, Západné Slovensko 15,800 € per inhabitant, Stredné Slovensko 14,100 € per inhabitant, Východné Slovensko 12,200 € per inhabitant. . BMI body mass index (*kg/m²*).
